# Peristaltic micropump using polyvinyl chloride gels with micropatterned surface

**DOI:** 10.1038/s41598-022-27226-3

**Published:** 2022-12-30

**Authors:** Tomoki Motohashi, Naoki Ogawa, Hideko Akai, Jun Shintake

**Affiliations:** 1 Department of Mechanical and Intelligent Systems Engineering, The University of Electro-Communications, 1-5-1 Chofugaoka, Chofu, Tokyo 182-8585 Japan; 2 Polymer Laboratory, Science and Innovation Center, Mitsubishi Chemical Co., Ltd., 1000 Kamoshida-cho, Aoba-ku, Yokohama-shi, Kanagawa 227-8502 Japan

**Keywords:** Materials for devices, Actuators, Fluidics

## Abstract

This paper presents a pump using polyvinyl chloride (PVC) gel. PVC gels are compliant, have a simple structure, and exhibit large deformation at voltages in the range of 100–1000 V, which make them suitable for micropumps. In this study, a PVC gel sheet with a surface pattern that enhances active deformation in the thickness direction was employed for the fabrication of a pump. To this end, the PVC gel sheet was sandwiched between three sets of anode and cathode electrodes, after which voltages were sequentially applied to these electrodes to generate a peristaltic deformation of the gel sheet, thus pushing the liquid and creating a one-directional flow. Various pumps were fabricated using PVC gel sheets with different surface patterns, and the pumps were characterized. The pumps exhibited an outline dimension of 35 mm × 25 mm with a thickness of 4 mm, corresponding to a total volume of 3.5 × 10^3^ mm^3^. The results revealed that the pump fabricated using a 174-μm-high pyramid-patterned gel sheet generated a flow rate of 224.1 µL/min at an applied voltage of 800 V and a driving frequency of 3 Hz. This observed value is comparable to or better than those of existing pumps based on smart materials.

## Introduction

Pumps are the fundamental element of fluid-driven systems, which are employed in a wide range of applications in medical, biology, chemistry, and robotics fields. In recent decades, the use of smart materials, which are materials that can deform themselves in response to external stimuli, has been widely investigated as a method of constructing pumps. This could be mainly attributed to their simpler structure compared to conventional pumps, which makes them scalable in size^1–4^. Accordingly, various pumps based on smart materials have been developed. These smart materials include shape memory alloys (SMA)^5–7^, piezoelectric ceramics (PZT)^8,9^, dielectric elastomers (DE)^10–13^, polyvinylidene fluoride (PVDF)^14^, ionic polymer metal composites (IPMC)^15–18^, and conductive polymers (CP)^19^.

As a smart material, polyvinyl chloride (PVC) gel exhibits promising features for pumps. PVC gels are normally synthesized by mixing PVC resins and liquid plasticizes (e.g., dibutyl adipate), and they typically have a sheet-like form^20–22^. When a PVC gel sheet is sandwiched between two electrodes and a potential difference is applied between them, the gel is attracted and deformed towards the positive electrode. This deformation is the result of charge injection from the negative side followed by the migration of plasticizer to the positive side^21–23^. PVC gels are soft, have a simple structure, and exhibit large deformations (e.g., 12% contraction strain^24^) and stresses (e.g., 5.26 kPa^24^) at voltages in the range of 100–1000 V^21,23^. The leakage current between the electrodes is said to be several tens of nanoamperes per square millimeter^22^. Owing to these features, PVC gels have been applied in various devices^22^, including an artificial muscle module^24^, gripper^25^, and human assistive device^26^. Although PVC gels have been applied in various devices, to the best of our knowledge, there are no studies on the use of PVC gels for the fabrication of pumps.

In this study, we develop PVC gel pumps to investigate the effectiveness of PVC gels in pumping devices. The developed pump was capable of generating continuous flow of liquid via the peristaltic deformation of the PVC gel sheet. The peristaltic deformation of the sheet was achieved by applying a voltage to multiple electrodes in the structure. The surface of the PVC gel sheet used in this study exhibited micropattern, which is expected to enable the efficient movement of liquid. Various PVC gel sheets with different micropatterns were fabricated and implemented in the pump, and their performance in terms of the flow rate as a function of the driving frequency of the peristaltic cycle at a fixed voltage was characterized.

## Results and discussion

**Figure 1:**
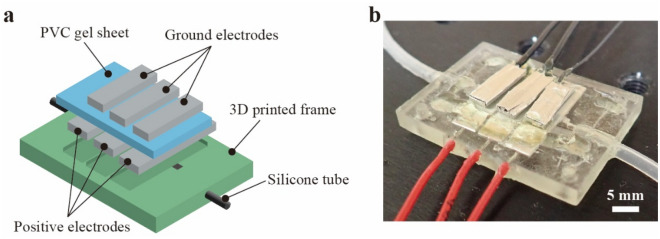
(**a**) Structure of the polyvinyl chloride (PVC) gel pump. (**b**) PVC gel pump developed in this study.

The entire structure of the PVC gel pump is shown in Fig. 1. It consists of a PVC gel sheet sandwiched between three sets of electrodes. The upper and lower electrodes were used as the positive and ground sides, respectively. The positive electrodes were fixed inside a 3D printed part, and a gel sheet, whose outer edge is glued to the 3D printed part, was placed on the positive electrode. The stickiness of the gel sheet ensured the attachment of the negative electrodes to the sheet top. The weight of the negative electrodes (0.34 g each) applies pre-stress to the gel sheet in the thickness direction so that the gel and positive electrodes are always in contact. Adjustment of pre-stress is possible by changing the weight of negative electrodes. Silicone tubes were connected to this structure to ensure the transportation of the liquid that passes through the domain between the positive electrodes and the gel sheet. The outline dimension of pump is 35 mm × 25 mm with a thickness of 4 mm, corresponding to the total volume of 3.5 × 10^3^ mm^3^. Further details on the fabrication process is presented in the “Methods” section.

**Figure 2:**
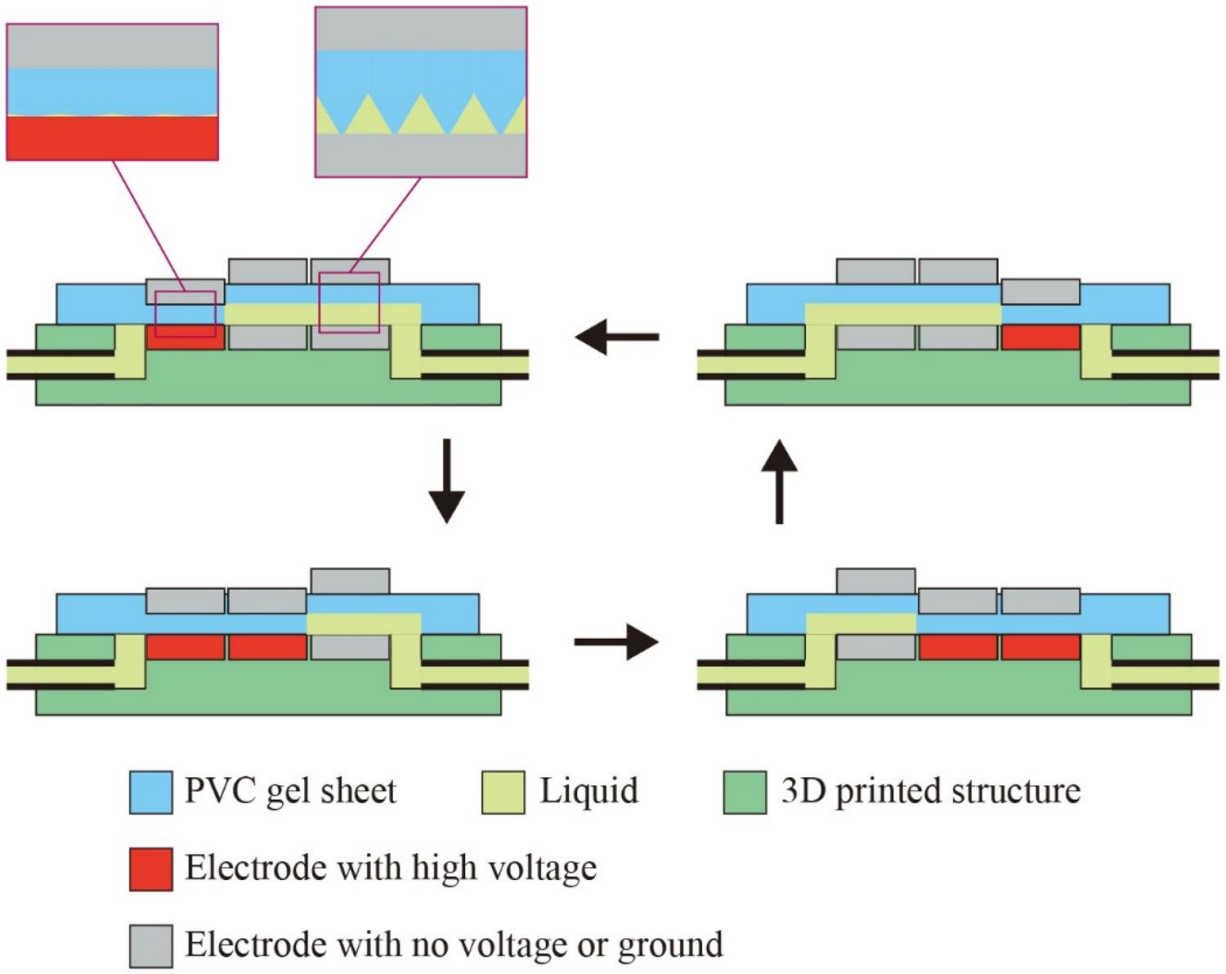
Working principle of the PVC gel pump.

The pump can generate the one-directional flow of the liquid when the electrodes are under cyclic activation. Figure 2 shows the activation of the electrodes during one cycle pumping, which consists of four patterns of electrode activation. The repetition of the same pumping cycle results in the continuous peristaltic deformation of the PVC gel sheet that transports the liquid in one direction.

**Figure 3:**
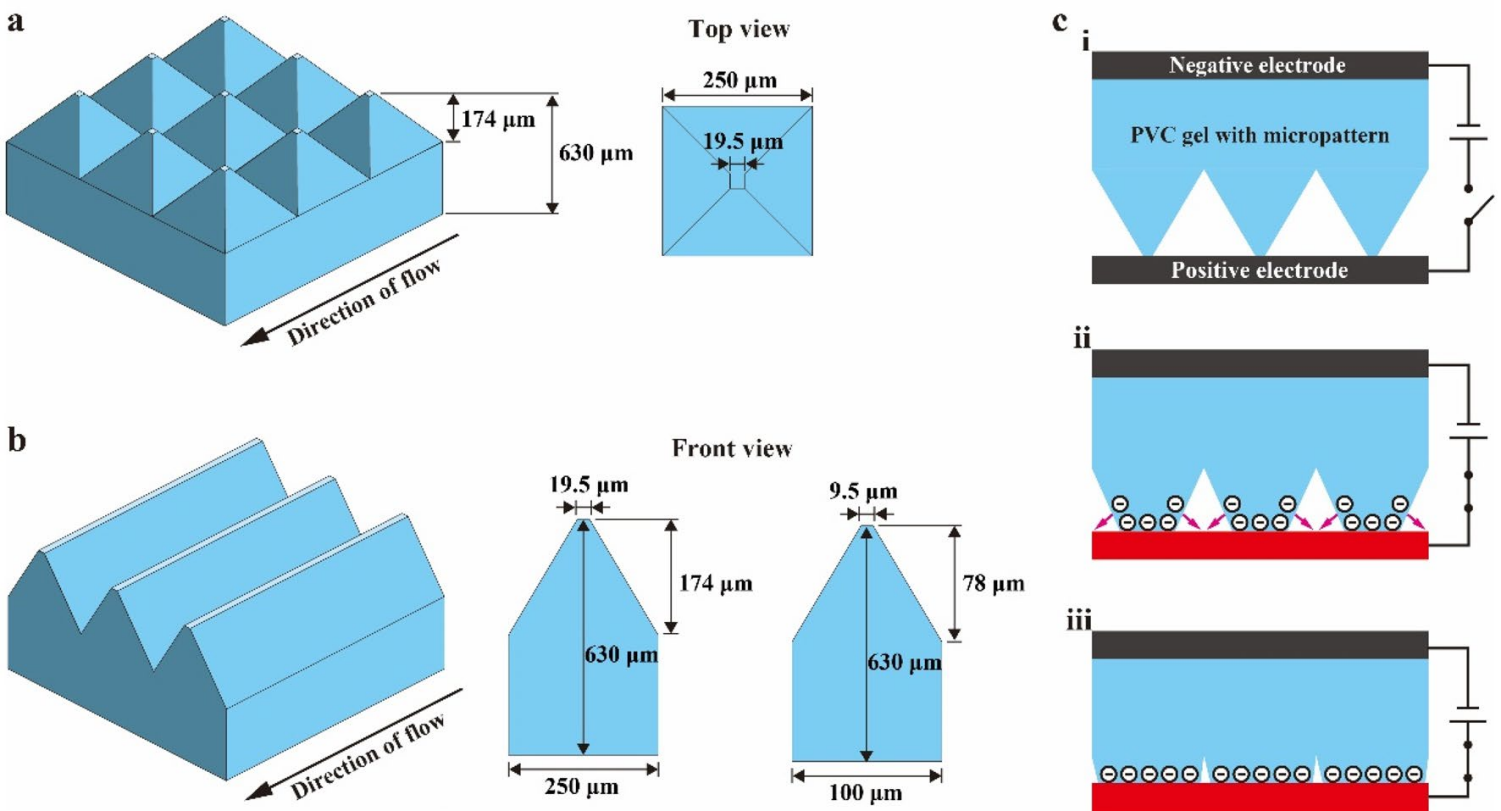
Geometry of the PVC gel sheets used in the pump. (**a**) Sheet with pyramid micropattern. (**b**) Sheet with ridge micropattern. (**c**) Actuation mechanism of a PVC gel sheet with micropattern. (i) The gel is sandwiched between positive and negative rigid electrodes. (ii) When a voltage is applied, the gel is deformed in a manner which flattens the vertices. (iii) The gel reaches an equilibrium state, where the entire structure exhibits a displacement change in the thickness direction.

The PVC gel pump was designed such that the gel sheet can push the liquid directly when it is in contact with the positive electrodes. Hence, it is difficult to take in liquid if there is no space between the gel sheet and the positive electrodes. This also results in a flat PVC gel sheet which does not produce flow because there is no route where the liquid can passthrough (no space between the gel sheet and the positive electrodes). Therefore, in this study, micropatterns were created on the surface of PVC gel sheet, and two patterns were considered: a pyramid pattern and ridge pattern. The gel sheets with these patterns are illustrated in Fig. 3 with their dimensions. The pyramid pattern (Fig. 3a) consisted of tetragonal pyramids with a height of 174 µm and a base of 250 µm. The ridge pattern (Fig. 3b) consisted of mountains with a width of 100 µm, and two different height (174 and 78 µm) were used for this pattern. The total height of every gel sheet was 630 µm. Different micropatterns and heights were considered in this study to investigate the effect of geometry on the pumping performance. The presence of the surface micropattern enabled the creation of a gap between the PVC gel sheet and the positive electrodes without precise positioning. In addition, it is expected to rectify the liquid passing through.

The actuation mechanism of the PVC gel sheet with micropattern is illustrated in Fig. 3c. In the pump configuration, the gel is sandwiched between positive and negative rigid electrodes, where the vertices of the pattern are in contact with the positive electrode. When a voltage is applied, electric charges are injected and attracted to the positive electrode. This causes the migration of plasticizer, resulting in a deformation in a manner flatten the vertices of the gel. The gel reaches an equilibrium state where the attractive force and elastic force of the gel are balanced. As the result, the entire structure exhibits a displacement change in the thickness direction. In this state, the gap between the gel and the positive electrode is minimized, meaning that if liquid is present, it is pushed in the horizontal direction.

**Figure 4:**
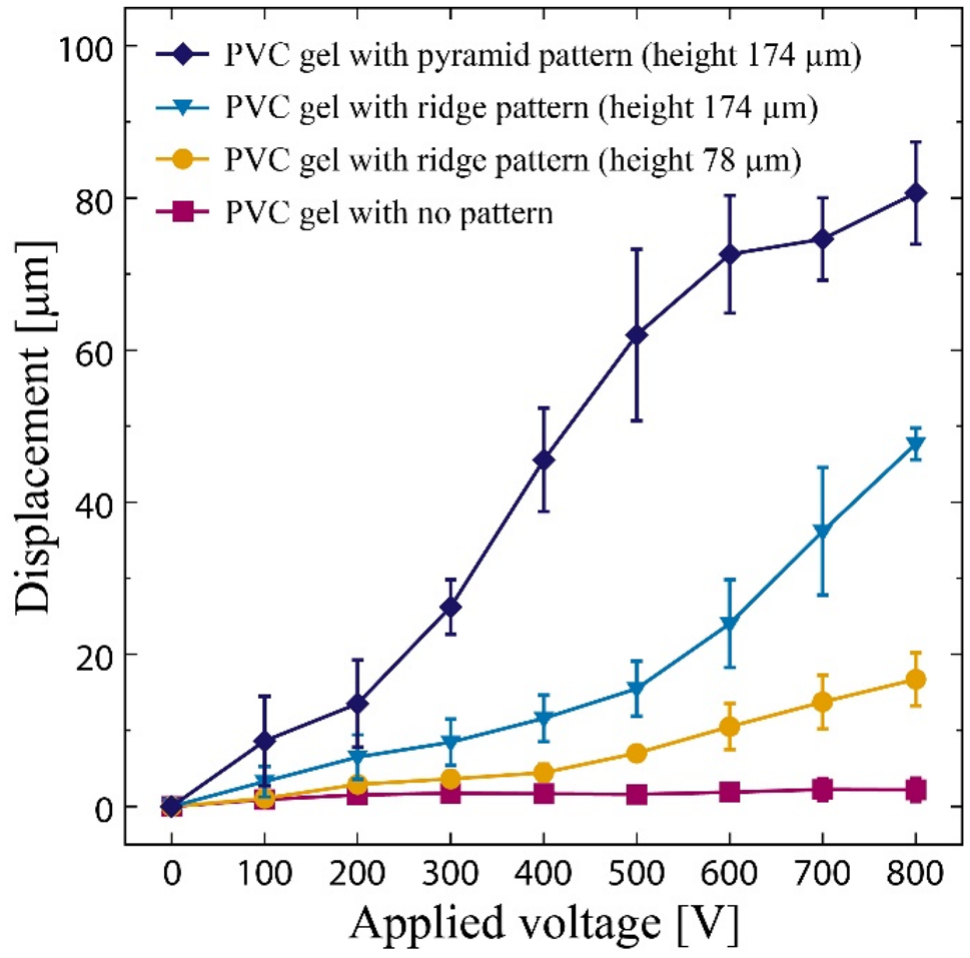
Displacement of the PVC gel sheets in the thickness direction as a function of the applied voltage. Every gel sheet was measured three times and the average was reported.

Moreover, the micropatterned PVC gel sheet was employed because it generates larger displacement in the thickness direction compared to options without micropattern. Figure 4 shows the plots of the displacement of the PVC gel sheets in the thickness direction as a function of the applied voltage. The tested gel sheets are those with the pyramid pattern (height 174 µm), ridge patterns (78 µm and 174 µm), and no pattern. Each of them is sandwiched between two rigid metal electrodes, and then displacement under application of voltage was measured (see “Methods” for more detail). The displacement of the patterned gel sheets was significantly higher than that of the non-patterned sheet, indicating its suitability for pumps that exploits the deformation in the thickness direction.

**Figure 5:**
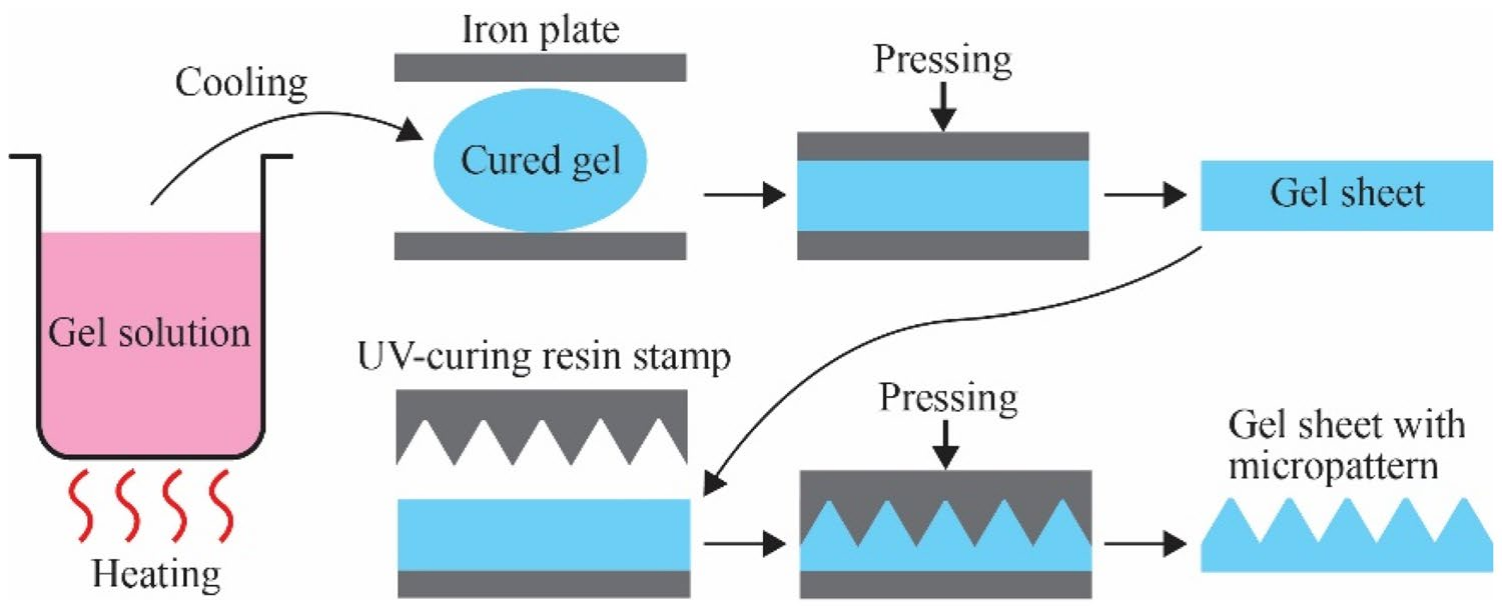
Fabrication process of the PVC gel sheets with micropattern.

The fabrication process of the micropatterned PVC gel sheet is summarized in Fig. 5 (see “Methods” for more detail). It starts from preparing a solution of PVC resin and a plasticizer (in this study, dibutyl adipate). After cooling, a cured PVC gel is obtained. Pressing the gel using heated iron plates results in a sheet form. The gel sheet is further pressed by an ultraviolet (UV)-curing resin stamp that has concavity with micropattern, resulting in the PVC gel with micropattern.

**Figure 6:**
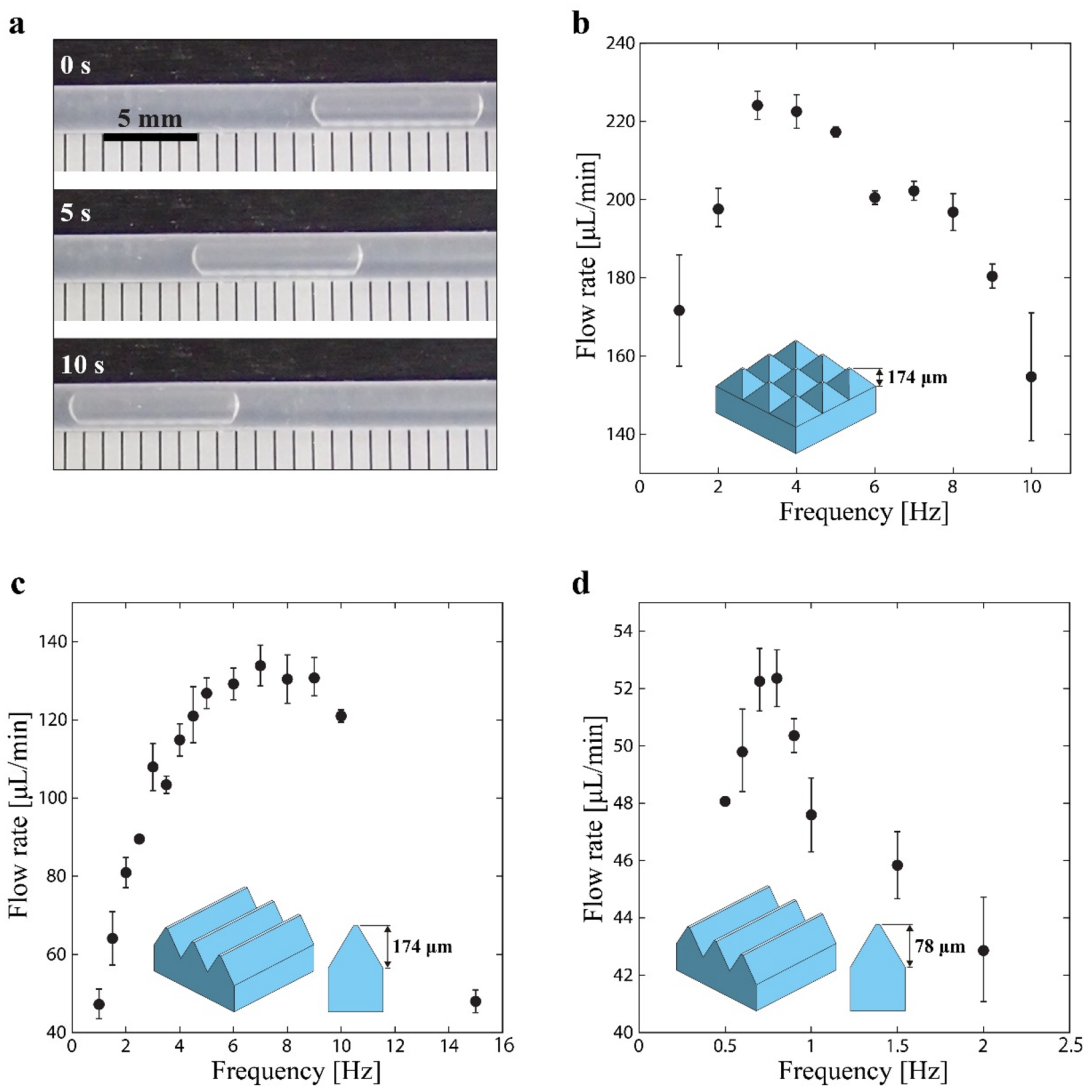
(**a**) Liquid flow generated by the PVC gel pump based on the observed bubble in the silicone tube. (**b**) Measured flow rate as a function of the driving frequency for the pump with a pyramid-patterned gel sheet (applied voltage 800 V). (**c**) Measured flow rate as a function of the driving frequency for the pump with a ridge-patterned gel sheet (applied voltage 800 V, height 174 µm). (**d**) Measured flow rate as a function of the driving frequency of the pump with ridge-patterned gel sheet (applied voltage 800 V, height 78 µm). For the measured flow rates, average of three times measurement is reported.

The movement of pumps fabricated using the aforementioned PVC gel sheets are shown in Supplementary Video S1. As shown in Fig. 6a, the transportation of the tested liquid (FC-43, 3M) can be observed by examining the movement of a bubble in the silicone tube (see also Supplementary Video S1). However, some backflow of liquid was observed during the cycle. This is because during the transformation from the upper right pattern to the upper left pattern shown in Fig. 2, there was a gap for the passage of the liquid before the full deformation of the PVC gel sheet in the upper left pattern. Based on the speed of a bubble and internal diameter of the tube, the flow rate generated from the pumps with a variation in the driving frequency (i.e., inverse of pumping cycle) at an applied voltage of 800 V was measured. Particularly, the flow rate was measured by determining the time a bubble travels a distance of 10 mm. This method, measuring the flow rate based on the speed of a bubble, also known as bubble time-of-flight, was employed by referring to various literatures^14,27,28^. The movement of a bubble was captured using a camera (TG-5, OLYMPUS), and the pump was driven using a high voltage power supply^29^. The magnitude of the voltage 800 V was chosen to remove any possibility of electrical breakdown while generating reasonable deformations. The waveform of the applied voltage was square, from which fast deformation of the gel was expected.

The measured flow rate of the pump with a pyramid pattern (height 174 µm) is plotted in Fig. 6b. With an increase in the driving frequency, the flow rate increased until it reached a peak value of 224.1 ± 5.4 µL/min at 3 Hz. With a further increase in the driving frequency beyond 3 Hz, the flow rate decreased. This trend is often observed in peristaltic pumps^22^, suggesting that our PVC pump functioned as expected. A similar trend was observed for the pumps with ridge patterns, as shown in Fig. 6c,d. The pump fabricated using the PVC gel sheet with a ridge height of 174 µm (Fig. 6c) achieved a peak value of 133.7 ± 6.4 µL/min at 7 Hz, which is 40% lower than that achieved by the one with the pyramid pattern. The peak flow rate of the pump fabricated using the gel sheet with a ridge height of 78 µm decreased to 52.3 ± 1.5 µL/min (0.8 Hz).

The different values of the peak flow rate and corresponding frequency may be attributed to several factors, the first being variation in the gap volume created by the micropatterns. The theoretical maximum displacement of the PVC gel sheet with the pyramid pattern (height = 174 µm) is 115 µm, which corresponded to a flow volume per pumping of 9.2 µL. For the gel sheets with ridge patterns of height 174 and 78 µm, the calculated maximum flow volume per pumping was 6.9 (max. disp. 82 µm) and 2.9 µL (max. disp. 33 µm), respectively. These values are consistent with the measured flow rates shown in Fig. 6 as well as the measured displacement in the thickness direction shown in Fig. 4. As for other factors, the PVC gel sheet with the pyramid pattern (height = 174 µm) has a larger displacement in the thickness direction (Fig. 4), so the overall amount of liquid to be extruded is larger. As the frequency is increased, the amount of deformation gradually decreases because the deformation of the gel cannot match the input. In this regard, the flow rate is determined by the multiplication of the frequency and the amount of deformation at that time. The frequency at which the flow rate peaks is 3 Hz for the pyramid pattern. For the ridge pattern with height of 174 µm, the overall flow rate is lower due to the relatively small displacement in the thickness direction. Alternatively, the peak frequency takes a higher value of 7 Hz for two potential reasons. The first is that a smaller displacement increases the frequency at which the maximum displacement can be leveraged, because the time required to reach that displacement is much shorter. The second is the ratio between the volume of the pattern and its surface area (the area in contact with the liquid). The larger the volume that can be actively deformed relative to the surface area, the easier it is to push the liquid out, thus increasing the frequency. In a 500 µm square unit on a PVC gel sheet, the 174 µm high ridge pattern has a surface area of 3.83 × 10^–5^ µm^2^ and a volume of 2.34 × 10^–7^ µm^3^, which gives a volume per unit surface area of 61.2 µm. This value is about two times higher than that of the pyramid pattern (29.9 µm), which correlates with the peak frequency that is about twice as high. For the ridge pattern with height of 78 µm, the volume per unit surface area is 22.5 µm suggesting that it requires a lower frequency than the pyramid pattern. In fact, the peak frequency of the ridge pattern with 78 µm height is 0.8 Hz. The peak flow rate is also less in proportion to the displacement in the thickness direction. However, as discussed above, the small displacement in the thickness direction contributes to an increase in the peak frequency, which is inconsistent. This suggests that there is a further factor that would reduce the frequency for the ridge pattern with 78 µm height.

We hypothesize that it is fluid friction due to the size of the pattern (the size of the flow channel). When the PVC gel is actively deforming in the pump and pushing liquid out, the flow created there is considered turbulent. In case of fully developed turbulence in the microfluidic channel, the fluid friction is expressed as the following nondimensional parameter^30^.

1 $$ \begin{array}{*{20}c} {f = 0.316/Re^{0.25} \quad (Re < 2 \times 10^{4} )} \\ \end{array} $$

Specifically, $f$ is called the Darcy friction factor. $Re$ is the Reynolds number given from the following equation.

2 $$ \begin{array}{*{20}c} {Re = \rho UD_{h} /\mu } \\ \end{array} $$

where $\rho$ is the density of liquid, $U$ the fluid velocity, $D_{h}$ the hydraulic diameter, and $\mu$ the dynamic (absolute) viscosity of liquid. Referring $\rho$ and $\mu$ from the datasheet of liquid used in this study (FC-43, 3 M)^31^, calculating $U$ based on the peak flow rate and the cross section of silicone tube on the output side, and then determining $Re$ with $D_{h}$ as the height of the pattern, the fluid friction factor is found to be 0.9 for the ridge pattern with height of 78 µm. The friction factor is 0.58 and 0.51 for the pyramid pattern (height = 174 µm) and the ridge pattern with 174 µm, respectively. Therefore, it is assumed that the fluid friction of the ridge pattern with height of 78 µm is about twice as large as that of the other patterns, which leads to additional flow resistance, and thus the frequency at which the peak flow rate is finally achieved is as low as 0.8 Hz. The above gives a guideline for designing PVC gel pumps. Namely, the larger the displacement in the thickness direction of the gel, the larger the active volume relative to the surface area, and the smaller the fluid friction, the higher the output will be in proportion to these factors.

We summarize the results obtained in this study and compared them to those of other pumps based on smart materials available in the literature. As shown in the table, the performance of our pump is comparable to or even better than those of devices based on other smart materials, indicating the high applicability and potential of PVC gel for pumps (Table 1).

**Table 1:** Comparison of the pumping performance of the PVC gel pump developed in this study and pumps based on other smart materials.

Actuator type	This work (PVC)	DE	CP	SMA	PZT	PVDF	IPMC
Principle of pump	Peristaltic	Peristaltic	Peristaltic	Peristaltic	Peristaltic	Diaphragm	Diaphragm
Materials flexibility	Soft	Soft	Soft	Rigid	Rigid	Soft	Soft
Flow rate per size (1/min)*^1^	6.4 × 10^–2^	3.0 × 10^–1^	4.2 × 10^–3^	1.2 × 10^–2^	4.2 × 10^–1^	4.8 × 10^–1^	1.9 × 10^–2^
Applied voltage (V)	800	4200	1.0	N/A	100	7200	2.0
Flow rate per voltage (µL/min V)	2.8 × 10^–1^	6.0 × 10^–1^	2.5	N/A	4.5	3.5 × 10^–3^	3.9 × 10^2^
Flow rate per size voltage (1/min V)*^1^	8.0 × 10^–5^	7.1 × 10^–5^	4.2 × 10^–3^	N/A	4.2 × 10^–3^	6.7 × 10^–5^	9.6 × 10^–3^
Continuous pumping	〇	×	×	×	〇	×	〇
Without valve	〇	〇	〇	〇	〇	〇	×
Reference		^11^	^19^	^6^	^8^	^14^	^16^

*^1^Volume is estimated from each reference.

## Concluding remarks

In this study, we developed a PVC gel pump, and confirmed the applicability of PVC gel to pumping devices. The experimental results revealed that the pump exhibited a peak flow rate of 224.1 µL/min, which is comparable to or even better than those of pumps based on other smart materials. In addition, the results suggested that the geometry of a micropattern is an important design parameter for PVC gel pumps and that the optimization of the pattern will improve the pumping performance.

Therefore, future work will focus on further characterization of PVC pumps under various geometries of surface micropattern, while considering factors regarding flow rate and driving frequency revealed from experimental results, activation sequence of the electrodes, and magnitude of the applied voltage as well as type of waveform. Acquiring actuation performance such as displacement and blocking force for different micropattern is expected to bring an insight for designing the PVC pumps by providing the relationship between actuation and pumping characteristics. To facilitate these experiments, a commercially available flow sensor will be employed instead of current method (bubble time-of-flight). In addition, the entire pump design could be modified. For example, increasing the active surface area, the area where the opposing electrodes overlap, will result in increased amount of the liquid per pumping cycle. Additionally, reducing the total thickness of the PVC gel sheets and modification of the material properties may enable the application of lower voltages, thus increasing the energy efficiency of the device.

## Methods

Briefly, polyvinyl chloride (PVC) (1700Z, Shindai-ichi vinyl) and dibutyl adipate (DBA) (Tokyo Kasei) were added into a separable flask at a weight ratio of PVC:DBA = 1:4. Next, the mixture in the flask was stirred and heated in an oil bath at 120 °C for 30 min at 90 rpm until a polymer gel was formed. Thereafter, the polymer gel was cooled and removed from the flask, and 3.6 g of the polymer gel was placed in a 70 mm dia. × 0.7 mm spacer and sandwiched between iron plates. Subsequently, the spacer was pressed using a hydraulic heating press machine at 150 °C and 2 MPa, after which the spacer was cooled to obtain a polymer gel sheet with a thickness of 630 µm. Thereafter, a UV-curing resin stamp with concave with micropattern was placed on the gel sheet, and the entire sample was placed into a vacuum heating press machine where the gel sheet was pressed at 150 °C and 0.1 MPa in vacuum state to create micropattern on the surface of the gel sheet. Different stamps were used to obtain the gel sheets with the pyramid and ridge patterns.

The main frame of the pump was fabricated using a 3D printer (Form3, Formlabs), and the 3D printing data was created using CAD (SolidWorks, Dassault Systèmes). Positive electrodes made of 1 mm-thick aluminum plate (20 mm-long, 5 mm-wide) were fixed inside the 3D printed frame, and these electrodes were wired through holes with a diameter of 0.7 mm drilled on the side of the frame. Holes with a diameter of 2.5 mm were also created where the silicone (outer diameter 2.5 mm, inner diameter 1.5 mm) tubes were connected. The PVC gel sheets were cut into outline dimensions of 20 mm × 16 mm and placed on top of the positive electrodes, and the edges of the gel sheet were bonded to the 3D printed main frame using UV-curable adhesive (BONDIC EVO, Spirit of Wonder). Subsequently, ground electrodes made of 1 mm-thick aluminum plate (16 mm-long, 5 mm-wide) were placed on the top of the PVC gel sheet. Wiring for every ground electrode was performed by attaching an electrical wire with a conductive tape.

In the measurement of active displacement in the thickness direction for the fabricated PVC gel sheets, every gel was punched into a circular shape with a diameter of 16.5 mm and sandwiched between two brass electrodes. The electrode on the positive side had a dimeter of 35 mm and thickness of 0.5 mm. The electrode on the negative side had a diameter of 10 mm and thickness of 2 mm. The positive and negative electrodes were placed on the bottom and top of the gel sheet, respectively. A laser displacement sensor (OPTEX-FA, CDX-L15) was used to measure the displacement of the top electrode (i.e., displacement of the gel sheet in the thickness direction) while applying voltage to the electrodes by a high voltage power supply^29^.

## Supplementary Information

Supplementary Video S1.

## Data Availability

All data that support the plot within this paper and other findings of this study are available from the corresponding author upon reasonable request.
